# Laser-Assisted Frenectomy Followed by Post-Operative Tongue Exercises in Ankyloglossia: A Report of Two Cases

**DOI:** 10.7759/cureus.23274

**Published:** 2022-03-17

**Authors:** Sowmiya Jaikumar, Lakshminarayanan Srinivasan, S.P.K. Kennedy Babu, D. Gandhimadhi, Manoj Margabandhu

**Affiliations:** 1 Department of Periodontology, Mahatma Gandhi Postgraduate Institute of Dental Sciences, Pondicherry, IND

**Keywords:** minor oral surgery, periodontal surgery, periodontics, diode laser, frenectomy, myofunctional therapy, laser, lingual frenectomy, ankyloglossia, tongue-tie

## Abstract

Ankyloglossia, commonly known as tongue-tie, is a developmental abnormality that may interfere in speech and articulation of lingual and sibilant sounds, due to the abnormal lingual frenal attachment. Lingual frenectomy severs the tie, however in adolescents and young adults, kinesthetic awareness, that is, the senses of position and movement of the tongue, needs to be increased. In such a scenario, tongue exercises lend a helping hand. Here, we discuss the benefits of this combined treatment modality in two cases diagnosed with ankyloglossia.

## Introduction

The term ankyloglossia was first described in the literature by Wallace in 1963. He defined ankyloglossia as a condition in which the tip of the tongue cannot be protruded beyond the lower incisor teeth because of a short lingual frenulum, often containing scar tissue [1]. Kotlow classified ankyloglossia into four, based on the free-tongue length. ‘Free-tongue’ is defined as the length of the tongue from the insertion of the lingual frenum into the base of the tongue to the tip of the tongue. If the free tongue length is greater than 16 mm, it is normal and clinically acceptable. When the free tongue length is 12-16 mm, it is considered mild ankyloglossia (class I), 8-11 mm is considered moderate ankyloglossia (class II), 3-7 mm is considered severe ankyloglossia (class III) and less than 3 mm is considered complete ankyloglossia (class IV) [2].

Ankyloglossia is associated with difficulties during suckling in infants, difficulties in speech associated with limited tongue movement in children [2]. But in adolescents and young adults, the difficulties in speech due to ankyloglossia may hinder social interactions and academic activities. The patients of this age group are self-aware of the difficulties they face [3]. The speech difficulties in ankyloglossia comprise difficulty in pronouncing r, s, j, ch, zh sounds and more effortful speech in general. Ankyloglossia may affect the development of maxillofacial complex and upper airway. Mouth breathing and snoring are also included as the potential implications of restricted tongue mobility [4].

The tongue with its normal range of movements acts as an excellent tool in maintaining oral hygiene due to its self-cleansing action. The sweeping motion of the tongue against food debris is restricted in ankyloglossia. Ankyloglossia may lead to gingival recession in the lingual aspect of lower anteriors [5]. Due to the above reasons ankyloglossia may compromise periodontal health. In many cases, ankyloglossia can be asymptomatic [6].

Lingual frenectomy is the primary treatment option for ankyloglossia. Laser-assisted frenectomy is advantageous over frenectomy with scalpel in many ways such as lesser intra-operative bleeding, lesser post-operative edema, better wound healing and relatively less pain [7]. Frenectomy alone may not bring out the desired results, especially in adolescents and adults as the tongue needs neuromuscular re-education. This necessary awareness which is lacking in the surgically treated tongue is gained by post-operative tongue exercises [8].

Here, we report two cases, a 14-year-old male patient and a 20-year-old male patient, diagnosed with ankyloglossia, managed by diode laser-assisted lingual frenectomy and adjunctive post-operative tongue exercises.

## Case presentation

Case 1

**Figure 1:**
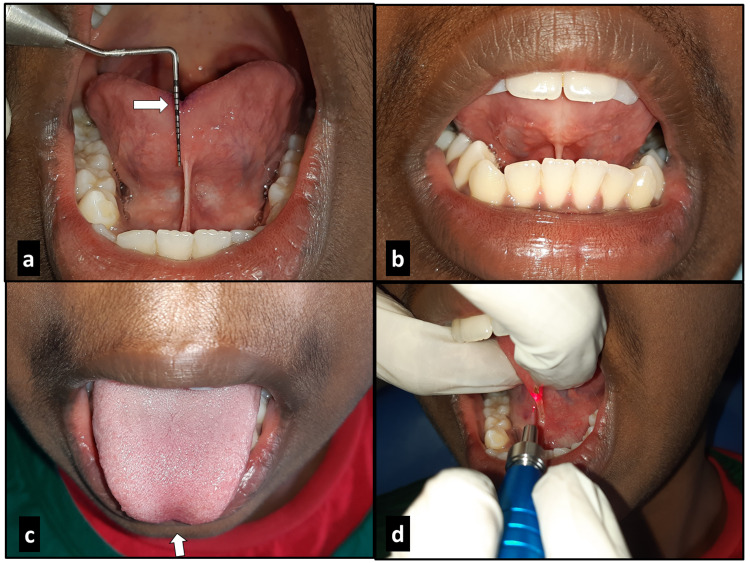
Case 1 – free tongue length of 11 mm (a); inability to curl the tongue tip towards the roof of the mouth (b); ‘W’ shaped appearance on protrusion of tongue – a classic feature of ankyloglossia (c); excision of lingual frenum with diode laser (d).

**Figure 2:**
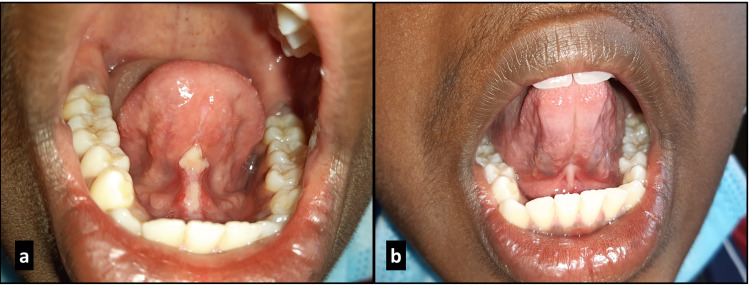
Case 1 – one week post surgery (a); five weeks post surgery (b).

A 14-year-old male patient reported to the Department of Periodontics with the chief complaint of difficulty in speech while articulating words with s, ch, zh sounds. General examination, oral examination, speech assessment were performed and the findings were recorded. Oral examination revealed a free tongue of 11 mm in length, which indicated moderate ankyloglossia (Kotlow’s class II) (Figure 1a). Figures 1, 2 present presurgical and postsurgical photographs to illustrate case 1.

Case 2

**Figure 3:**
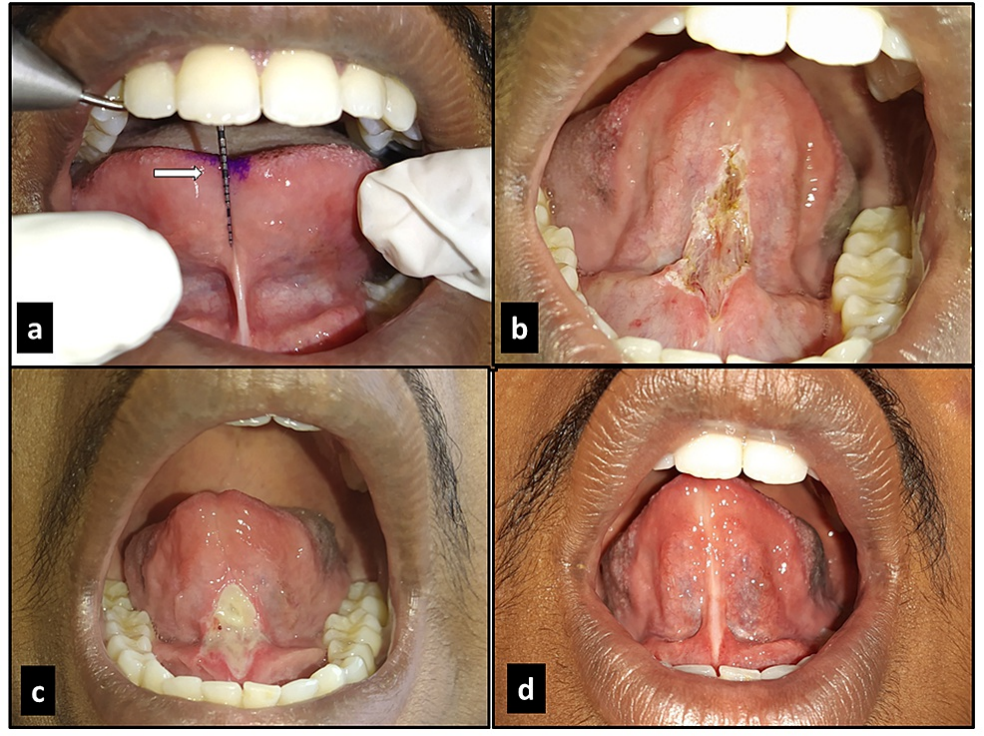
Case 2 – free tongue length of 10 mm (a); after frenectomy with diode laser (b); one week post surgery (c); five weeks post surgery (d).

A 20-year-old male patient reported to the Department of Periodontics with the chief complaint of difficulty in speech and in articulating certain words with r, zh sound. General examination, oral examination and speech assessment were performed and the findings were recorded. Oral examination revealed a free tongue of 10 mm in length, which indicated moderate ankyloglossia (Kotlow’s class II) (Figure 3a). Figure 3 illustrates case 2.

In both the cases, on protrusion of the tongue, the tip of the tongue formed a W-shaped appearance (Figure 1c). The speech assessment suggested difficulty in pronunciation of sounds like s, z, ch and zh (sibilant consonants in which tip of the tongue is brought near the roof of the mouth) and furthermore difficulty in pronunciation of the r sound (rhotic consonant where the tongue-tip is curled towards the roof of the mouth) (Figure 1b). The patients were diagnosed with ankyloglossia (Kotlow’s class II) and the treatment plan included lingual frenectomy to relieve the frenum facilitating normal tongue movements and post-operative tongue exercises to increase kinesthetic awareness of the full range of movements the tongue can perform.

Under infiltration of local anesthetics, 2% lignocaine in 1:80,000 adrenaline, the lingual frenum was excised by diode laser (wavelength of 980 nm, power of 2 watts and in continuous contact mode) (Figure 1d). Bleeding during the procedure was almost negligible (Figure 3b). Post-operative pain and discomfort were minimal. Post-surgical instructions were given and analgesic (tablet paracetamol 500 mg) was prescribed, to be taken if required. The patients were followed up after one week (Figures 2a, 3c).

The wound was healing satisfactorily and post-operative tongue exercises were taught and initiated with instructions to execute the exercises thrice a day for four weeks. On review after four weeks, a considerable increase in tongue mobility was noted and patients were able to articulate words with r, s, z, ch and zh sounds more confidently with ease (Figures 2b, 3d).

## Discussion

Normal tongue movements are significant in suckling, swallowing, chewing and above all speech. The position of the tongue and its attachment can affect the position of teeth and periodontal tissue [2]. Abnormalities in tongue movements can have many etiological factors, including orofacial dyskinesia, hypoglossal nerve dysfunction, among which ankyloglossia is one of the prominent reasons for restricted tongue movement.

Suspected etiologies of ankyloglossia

Though the exact etiology of ankyloglossia is unknown, it is considered to be a developmental anomaly in which both environmental and genetic factors play a role. In some cases, ankyloglossia had been associated with syndromes such as X-linked cleft palate syndrome, Kindler syndrome, Opitz syndrome and van der Woude syndrome [5]. Similarly, speech difficulties have a wide range of etiologies ranging from neural, cerebral causes to hearing disabilities and intellectual impairments. Therefore, it is necessary to rule out other causes and syndromes before diagnosing ankyloglossia as the cause for difficulty in speech.

Surgical management

Management of ankyloglossia is usually surgical where the abnormal frenal attachment is relieved by frenectomy, frenotomy or frenuloplasty. Frenectomy is defined as complete excision and removal of the whole frenum [5]. Conventional frenectomy with scalpel may result in considerable intra-operative bleeding and will need closure of the surgical site with sutures. Post-operative pain and swelling may be seen as a consequence. In contrast, frenectomy with the help of laser results in minimal bleeding and sutures are not necessary, and post-operative pain is negligible. Due to such factors, laser-assisted frenectomy has better patient compliance [6,7]. In children, adolescents and young adults dental anxiety is common. Laser-assisted procedures are less time-consuming with minimal discomforts, therefore help in overcoming anxiety. The above-mentioned age groups and anxious patients can be treated efficiently with laser surgeries [7].

In the cases discussed above, a diode laser (980 nm) with a power of 2 watts was employed. The fiber-optic tip (400 µm) was initiated before use. The frenum was excised in continuous mode, with the tip contacting the frenum in brushing stroke, each stroke lasted for one second and the strokes were repeated until the complete frenum was excised.

Post-operative healing was compared among different methods and the results indicated healing was uneventful in laser and electrocautery methods, whereas post-operative edema and pain were evident in the scalpel method. In an in-vitro analysis, histologically, healing was complete in one month in all the three methods and the skeletal muscle fibers were better arranged in healing after the scalpel method. Clinically, in the immediate post-operative phase, laser and electrocautery exhibited faster healing since manipulation of tissues was better [6].

Role of orofacial myofunctional therapy or active kinesthetic therapy

The role of post-operative tongue exercises in the management of ankyloglossia is often underestimated. Post-operative exercises are not meant to improve speech, rather they increase the kinesthetic awareness of the tongue. When normal tongue function is restricted by ankyloglossia, the other muscles of the oral cavity, i.e., the stomatognathic system, compensate for it to an extent. Therefore, after surgical correction of ankyloglossia, the tongue has no “muscle memory” of how it should function without restriction. This is where orofacial myofunctional therapy, incorporated in the form of rehabilitative tongue exercises, helps in re-educating the tongue [8]. The ability to elevate the tongue increased following adjunctive kinesthetic therapy [5]. The exercise protocol recommended by Tecco et al. [8] was explained to the patient and the patient’s cooperation was ensured (Table 1).

**Table 1:** Post-operative tongue exercises instructed and demonstrated to the patient. *The exercise protocol is a simplified version, based on the recommendations by Tecco et al. (2015) [8].

S. no.	*The following exercises are repeated 15 times, thrice a day for four weeks. The patient is encouraged to perform these exercises in front of a mirror, for better compliance.
1	The tongue is protruded as much as possible.
2	The tip of the tongue is moved forwards and upwards as much as possible, trying to touch the tip of the nose.
3	The tongue is protruded outside the mouth and rotated to perform circular movements, first clockwise and then anti-clockwise.
4	The tip of the tongue is positioned on the incisive papilla and in that position, the mouth is opened and closed.
5	The above exercise is repeated with the tongue position secured in the mid-palate and posterior palate.
6	The entire tongue is pushed against the palate, and a vacuum is created by sucking the air. Now, the mouth is opened slowly to the maximum extent possible, until the tongue gets released from its position.

In the cases discussed, tongue mobility one week after surgery had improved, however, was limited. After initiating the practice of tongue exercises, a significant improvement in tongue mobility was noted. Free tongue length calibrated five weeks after surgery was greater than 16 mm in contrast to free tongue length initially calibrated which was 11 mm and 10 mm, respectively. And the patients were able to perform protrusive and other such tongue movements with more ease and comfort, which were in concordance with the results obtained by Tecco et al. [8].

## Conclusions

Thus, managing ankyloglossia with the combination of surgery and post-operative exercises - orofacial myofunctional therapy - is shown to yield better and desired results. This is especially true when treating adolescents and young adults, where re-educating the tongue, of the missing muscle memory, becomes significantly important.

The laser-assisted frenectomy resulted in minimal post-operative pain and discomfort, thus making the patient physically and psychologically ready to follow the exercise pattern one week post-surgery. The results of such rehabilitative exercises depend upon the patient's adherence to the exercise protocol. Speech therapy can be initiated if deemed necessary. Further research and randomized control trials are needed to evaluate this combined treatment plan.
